# Association between sarcopenia and osteoporosis: the cross-sectional study from NHANES 1999–2020 and a bi-directions Mendelian randomization study

**DOI:** 10.3389/fendo.2024.1399936

**Published:** 2024-10-08

**Authors:** Yuan Zhu, Qingyue Zeng, Yi Shi, Yu Qin, Simin Liu, Yuhao Yang, Yu Qiu, Mengjia Pan, Zhenmei An, Shuangqing Li

**Affiliations:** ^1^ General Practice Medical Center, West China Hospital, Sichuan University, Chengdu, Sichuan, China; ^2^ Department of Endocrinology and Metabolism, West China Hospital, Sichuan University, Chengdu, Sichuan, China; ^3^ General Practice Medical Center, National Clinical Research Center for Geriatrics, West China Hospital, Sichuan University, Chengdu, Sichuan, China

**Keywords:** sarcopenia, NHANES, MR, ALM, osteoporosis

## Abstract

**Background:**

Osteoporosis (OP) and sarcopenia are prevalent musculoskeletal conditions among the elderly. Nevertheless, the causal relationship between sarcopenia and OP remains a subject of controversy and uncertainty. In this study, we employed cross-sectional analysis and Mendelian randomization (MR) to investigate the intricate relationship between sarcopenia and OP.

**Methods:**

The cross-sectional study utilized data from the National Health and Nutrition Examination Survey (NHANES) spanning 1999-2020, which involved in 116,876 participants. It assessed the correlation between sarcopenia, osteoporosis (OP), and bone mineral density (BMD) using Chi-square tests, T-tests, and a multiple logistic regression model. Additionally, we conducted Mendelian randomization (MR) analysis to investigate the causal effects of sarcopenia-related characteristics (ALM) on OP. We employed IVW, sensitivity analysis, heterogeneity testing, and other methods for MR. The ALM data was sourced from the UK Biobank (n=450,243), while the aggregated data on OP was obtained from GWAS statistics (n=53,236).

**Results:**

In this cross-sectional analysis, we observed that in the multivariate logistic regression model, without adjusting for any variables, OP emerged as a risk factor for sarcopenia [OR 95% CI = 1.90 (1.13-3.18), P = 0.02]. Following adjustments for gender, age, BMI, and biochemical variables, OP retained its status as a risk factor for sarcopenia [OR 95% CI = 3.54 (1.91-6.54), P < 0.001]. Moreover, after accounting for all variables, OP emerged as an independent risk factor for sarcopenia [OR 95% CI = 4.57 (1.47-14.22), P = 0.01].In the MR analysis, we uncovered that femoral neck BMD (FN BMD), lumbar spine BMD (LS BMD), and forearm bone mineral density (FA BMD) exerted a direct causal influence on ALM [FA BMD: OR 95% CI = 1.028 (1.008, 1.049), p = 0.006; FN BMD: OR (95% CI) = 1.131 (1.092, 1.170), p = 3.18E-12; LS BMD: OR (95% CI) = 1.080 (1.062, 1.098), p = 2.86E-19].

**Conclusion:**

Our study has revealed a positive correlation between OP and the prevalence of sarcopenia. It suggests a potentially robust causal relationship between OP and sarcopenia. Notably, OP appears to be associated with a higher likelihood of losing ALM, and a significant loss of ALM may contribute to a decline in LS BMD.

## 1 Introduction

Osteoporosis (OP) is characterized by a reduction in bone mass and deterioration of bone tissue microstructure, leading to decreased bone strength and an increased risk of fractures. These fractures pose a significant public health challenge, contributing to morbidity, functional impairment, reduced quality of life, and even mortality (1). Globally, approximately 18.3% of the population is affected by OP, with a higher prevalence in women (23.1%) compared to men (11.7%) (2). From an etiological perspective, OP is classified into two main types: primary OP and secondary OP. Primary OP is associated with age-related changes and typically occurs in individuals aged 50 years or older (3). The most common form of primary OP is postmenopausal osteoporosis, which results from decreased estrogen secretion following menopause. Conversely, secondary OP arises from specific medications and medical conditions that lead to decreased bone mineral density (BMD) (4). The pathogenesis of OP involves various pathogenic factors, including the gut microbiome, autophagy, abnormal iron metabolism, aging, and stress (5). These factors interact in a complex network to contribute to the pathological process of OP. Aging, a major risk factor for OP, can affect gut microbiota composition, cellular autophagy, and iron metabolism, thereby inducing bone marrow mesenchymal stem cell senescence in the OP pathological process. This is mediated through the regulation of immune responses, metabolic pathways, mitophagy, p53 expression, and reactive oxygen species production (6). That means osteoporosis, like sarcopenia, is an age-related disease.

Sarcopenia is characterized by a widespread and progressive loss of skeletal muscle mass and function (7). Its pathogenesis and pathophysiology are multifaceted, involving changes in skeletal muscle protein metabolism, hormonal processes, neurophysiology, inflammation, vascularization, and mitochondrial function (8). Coined by Rosenberg in 1988, the term originally referred to the gradual decline in lean muscle mass, creatinine excretion, basic metabolic rate, and muscle strength, starting around the age of 20-30 and persisting throughout aging (9). By 2010, the definition of sarcopenia evolved to encompass reduced muscle mass, strength, and physical performance (10). This age-related condition is associated with an increased risk of disability, reduced functional independence (7, 9, 11) and OP (12). The prevalence of sarcopenia varies across populations, with incidence rates ranging from 5% to 50% in individuals aged 65 and above (13). Sarcopenia typically emerges in the seventh decade, affecting 5-13% of individuals, a figure that may rise to 11-50% by age 80. It is anticipated that by 2050, over 500 million elderly people will be affected. Sarcopenia is linked to numerous adverse health outcomes. A recent quantitative analysis of 30 studies and 40,000 participants demonstrated that sarcopenic individuals face a 1.89-fold increased risk of falls and a 1.71-fold increased risk of fractures (10). Sarcopenia is associated with many adverse health outcomes. Recently, a quantitative analysis of 30 studies and 40,000 participants showed that sarcopenic individuals have a 1.89-fold and 1.71-fold increased risk of falls or fractures, respectively (14). Consequently, an increasing number of clinicians are emphasizing the prevention and intervention of factors associated with sarcopenia.

The coexistence of osteoporosis and sarcopenia has been recently defined as osteo-sarcopenia (15) or sarco-osteoporosis (16), affecting roughly 37% of the elderly population, thus posing a significant global health burden (17). Osteo-sarcopenia or sarco-osteoporosis is diagnosed in individuals meeting criteria for both sarcopenia and either osteopenia or osteoporosis. Research on osteo-sarcopenia commonly employs DXA (18), computed tomography, or magnetic resonance imaging, although methods like deuterated creatinine may offer more precise measurements of total body muscle mass (19). Patients with cachexia, compared to those with simple sarcopenia or osteoporosis, tend to be older, with lower grip strength, lower T scores, poorer balance, and reduced functional capacity (20). The adverse outcomes associated with the combination of OP and sarcopenia are more severe than those of either condition alone (21, 22).

To investigate the relationship between sarcopenia and OP, we initially conducted an observational study using data from the US population based on the National Health and Nutrition Examination Survey (NHANES) database. Additionally, we performed a bidirectional two-sample Mendelian randomization (MR) analysis to discern the causal impact of sarcopenia on the risk of OP from a genetic variation perspective. This study lays a theoretical foundation for the early identification and prevention of individuals with sarcopenia and/or OP.

## 2 Materials and methods

### 2.1 Epidemiological cross-sectional study design and data source

The data for this study were sourced from the NHANES, a cross-sectional survey program designed to evaluate the health and nutritional status of both adults and children in the United States. Additionally, NHANES received approval from the Institutional Review Board of the National Center for Health Statistics (NCHS), a division of the Centers for Disease Control and Prevention (CDC) responsible for generating vital health statistics for the nation (23).

**Figure 1:**
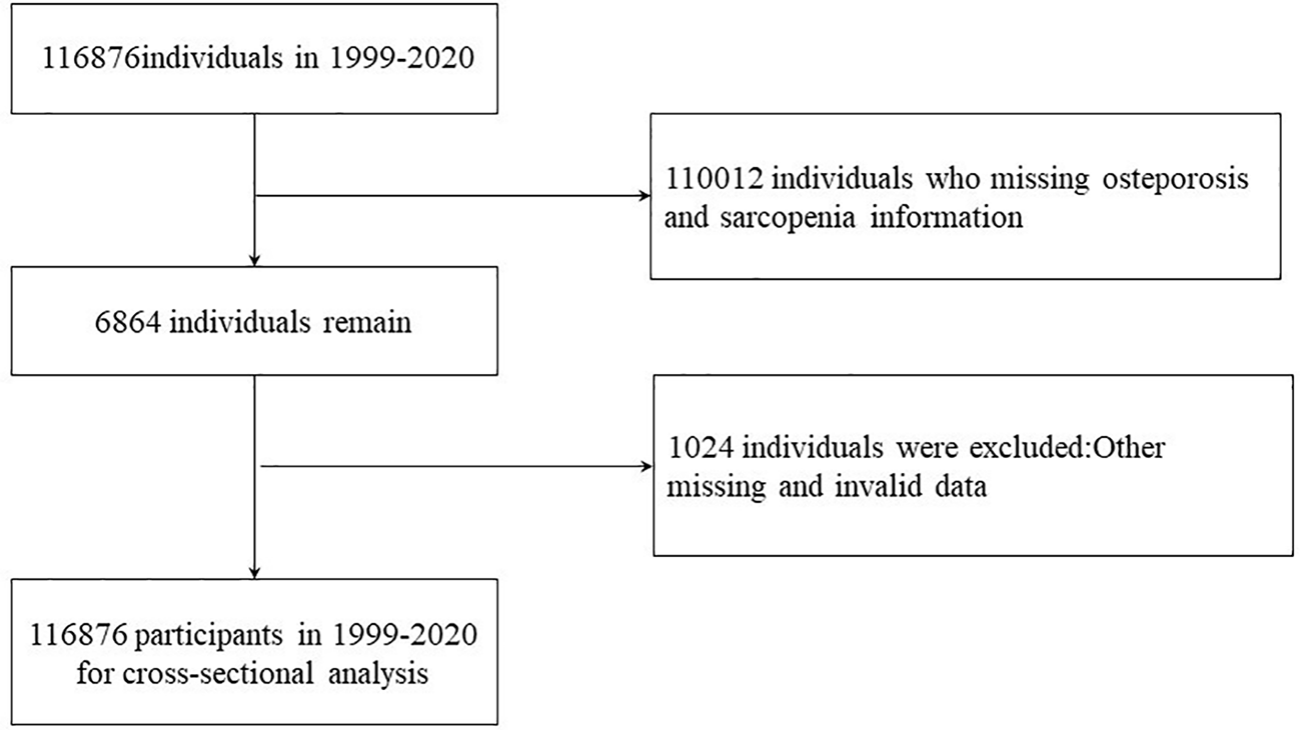
Study flowchart. NHANES, National Health and Nutrition Examination Survey.

Our research integrated information from the NHANES database spanning from 1999 to 2020, encompassing eleven survey cycles. In total, 116,876 participants completed demographic surveys, underwent physical examinations, participated in laboratory tests, and responded to health condition questionnaires. Exclusion criteria were applied as follows (1): Instances with missing data on osteoporosis and sarcopenia (n=110,012); (2) Cases lacking information on body mass index (BMI) and other covariates (n=1,024). Ultimately, a total of 5,840 participants were included in this analysis (**Figure 1**).

### 2.2 All variables included in the cross-sectional analysis

In NHANES, the primary study indices, BMD and appendicular Lean Mass (ALM), were measured using dual-energy X-ray absorptiometry (DXA). Sarcopenia was defined with cut-off values of 0.789 kg/m² for men and 0.512 kg/m² for women (24). OP was confirmed based on responses to the Medical Conditions Questionnaire (MCQ). Covariates included age, gender, race, family income-to-poverty ratio (PIR), weight, height, BMI, waist circumference (WC), and laboratory tests including ALT, AST, total bilirubin, albumin, total protein, creatinine (Cr), blood urea nitrogen (BUN), Cr/BUN ratio, uric acid (UA), total cholesterol (TC), triglycerides (TG), and high-density lipoprotein (HDL).

### 2.3 Sources of bidirectional MR

The UK Biobank stands as a significant biomedical database and research asset, housing comprehensive genetic and health data from over half a million participants in the United Kingdom. ALM, a crucial metric for assessing muscle mass in older adults, was computed by aggregating the fat-free mass of 450,243 participants from the UK Biobank cohort. This calculation was adjusted for appendicular fat mass, age, and other relevant covariates (25). This metric serves as a valuable tool for identifying clinically significant sarcopenia (26).

In clinical practice, femoral neck bone mineral density (FN BMD), lumbar spine bone mineral density (LS BMD), and forearm bone mineral density (FA BMD) are extensively utilized for predicting OP (27). Through the synthesis of genetic factor data from GWAS statistics, collected from 53,236 participants’ BMD, we endeavored to pinpoint factors associated with OP, while adjusting for gender, age, and weight (28). The populations analyzed were predominantly of European descent, with accessibility facilitated through the IEU Open GWAS database via their respective IDs.

### 2.4 Statistical analysis of the cross-sectional study

In the cross-sectional statistical analysis, baseline characteristics of all participants were presented as mean ± standard deviation (SD) for continuous variables and as N (%) for categorical variables. To enhance individual participant representation, we applied probability sampling weights, accounting for survey non-response, oversampling, post-stratification, and sampling errors. The Examination Center (MEC) utilized sample weights to address data oversampling, filling in missing values with either the mean or mode of the sample, given that the percentage of missing data was below 10%.

The normality of distribution for each continuous variable was assessed using the Shapiro-Wilk test and the Kolmogorov-Smirnov test. Initially, the T-test and chi-square test were employed to examine differences in baseline attributes between participants with and without OP. Subsequently, to further explore the relationship between independent and dependent variables, multiple regressions were conducted. We performed weighted multiple logistic regression analyses, adjusting for various confounding factors, and constructed three models to estimate the odds ratio (OR), p-value, and 95% confidence interval (CI) between BMD, OP, and sarcopenia risk. Furthermore, we investigated the correlation between bone density stratification (quartiles) and sarcopenia across different genders and races. Significance was determined at P < 0.05 for all tests. This study utilized R software version 4.3.1 for statistical analysis.

### 2.5 Bidirectional mendelian randomization analysis

**Figure 2:**
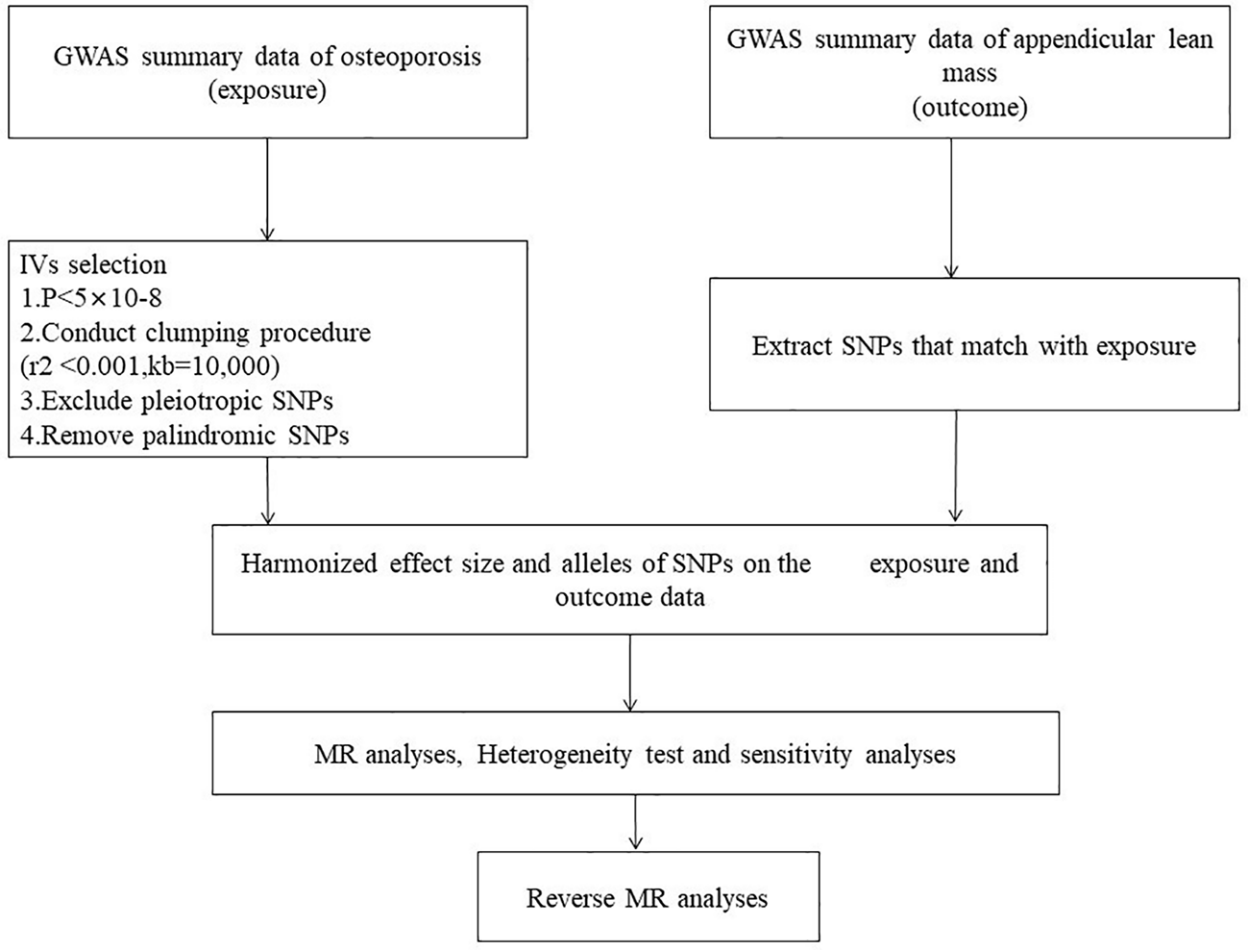
Mendelian randomization Flowchart. Analysis of causality between sarcopenia-related traits and osteoporosis.

Bidirectional two-sample MR analysis is a method that leverages genetic instrumental variables (IV) to assess the causal relationship between an exposure and an outcome. Three hypotheses guided the MR analysis (1): The IV is associated with the exposure factor, (2) The IV is independent of confounding factors, and (3) The IV influences the outcome solely through the exposure factors (**Figure 2**). This bidirectional MR analysis was conducted in two steps: In the first step, Osteoporosis (OP) was examined as the exposure, while Sarcopenia (SP)-related traits were investigated as the outcome. In the second step, this relationship was reversed.

The inverse variance weighted (IVW) method, a primary statistical technique, was employed to assess the bidirectional relationship between sarcopenia and OP. Following the three assumptions for Mendelian randomization analysis, single-nucleotide polymorphisms (SNPs) independently associated with the exposure at the genome-wide significance level (p < 5 × 10^-8) were selected as instrumental SNPs (clumping r^2 = 0.001 and kb = 10,000) (29). The IVW method is considered the most accurate approach for evaluating causal connections in the absence of compelling evidence suggesting directional pleiotropy (P-value for MR-Egger intercept > 0.05) (30). However, we relaxed the criteria to 1 × 10^-6 when selecting instrumental variables related to forearm bone mineral density (FA BMD), and only three SNPs were chosen.

To assess the robustness of our findings, various sensitivity analyses were conducted. Heterogeneity among instrumental variables was evaluated using Cochran’s Q statistic. Additionally, the MR pleiotropy test was employed to conduct MR Egger analysis, yielding intercept values to assess horizontal pleiotropy. Statistical significance was defined as P < 0.05. The MR tests were performed using the R packages “TwosampleMR”, “Mendelian Randomization”, and “MRPRESSO” in the R statistical software (Version 4.3.1).

## 3 Results

### 3.1 Epidemiological cross-sectional study observation and analysis

Participant characteristics were extracted from the NHANES 1999-2020 database, ultimately comprising 5,840 individuals in our study. **Table 1** provides an overview of the baseline characteristics of the study population across eleven cycles. The average age of the 5,840 participants was 40.72 years, with 49.29% being female. According to AWGS criteria, 599 (10.3%) and 5,241 (89.7%) participants were diagnosed with sarcopenia and non-sarcopenia, respectively. Variables such as age, gender, race, height, weight, PIR, total bilirubin, total protein, albumin, creatinine, blood urea nitrogen, creatinine-urea nitrogen ratio, uric acid, LS BMD, pelvis-BMD, total BMD, and sarcopenia exhibited significant statistical differences between individuals with and without sarcopenia (P < 0.05).

**Table 1:** Baseline characteristics of the research population with and without sarcopenia.

Characteristic	Total	Non-sarcopenia (n=5241)	Sarcopenia (n=599)	P-Value
Age	40.72 ± 0.41	41.55 ± 0.40	34.13 ± 0.99	<0.0001
Gender (%)				
Female Male	49.29 50.71	50.80 49.20	37.32 62.68	<0.0001
Race (%)				
Mexican American Non-Hispanic Black Non-Hispanic White Other Hispanic Other Race Height(cm) Weigh (kg) BMI (kg/m2) Waist-circumference(cm) PIR ALT AST Total-bilirubin Total-Protein Albumin Albumin-urine UACR Creatine Blood urea nitrogen Cr/BUN Uric-acid TG HDL TC LS BMD Pelvis-BMD Total BMD	9.24 10.58 67.82 4.95 7.42 167.14 (0.25) 75.70 ± 0.57 26.81 ± 0.17 92.98 ± 0.48 3.17 ± 0.06 25.12 ± 0.36 25.20 ± 0.30 11.54 ± 0.13 70.88 ± 0.15 42.82 ± 0.10 31.30 ± 3.29 26.83 ± 2.50 76.45 ± 0.47 4.32 ± 0.03 19.31 ± 0.16 307.47 ± 1.62 1.65 ± 0.02 1.41 ± 0.01 5.00 ± 0.02 1.01 ± 0.00 1.25 ± 0.00 1.12 ± 0.00	7,89 11.21 69.50 4.38 7.02 168.97 ± 0.21 76.93 ± 0.57 26.76 ± 0.18 93.18 ± 0.48 3.23 ± 0.06 25.26 ± 0.39 25.26 ± 0.32 11.69 ± 0.13 70.80 ± 0.15 42.88 ± 0.11 29.43 ± 3.48 25.25 ± 2.67 77.93 ± 0.46 4.36 ± 0.03 19.57 ± 0.17 309.09 ± 1.64 1.63 ± 0.03 1.41 ± 0.01 5.01 ± 0.02 1.03 ± 0.00 1.27 ± 0.00 1.14 ± 0.00	19.93 5.59 54.49 9.41 10.58 152.63 ± 0.59 66.03 ± 1.49 27.22 ± 0.45 91.35 ± 1.22 2.66 ± 0.11 24.02 ± 0.68 24.73 ± 0.46 10.36 ± 0.26 71.50 ± 0.23 42.28 ± 0.14 46.07 ± 12.46 39.28 ± 8.77 64.75 ± 1.17 3.96 ± 0.06 17.24 ± 0.24 294.70 ± 4.10 1.78 ± 0.08 1.37 ± 0.02 4.92 ± 0.05 0.86 ± 0.01 1.09 ± 0.01 1.00 ± 0.01	<0.0001 <0.0001 <0.0001 0.320 0.140 <0.0001 0.110 0.310 <0.0001 <0.01 <0.0001 0.220 0.140 <0.0001 <0.0001 <0.0001 0.001 0.100 0.060 0.110 <0.0001 <0.0001 <0.0001
Osteoporosis (%)				
Normal Osteopenia Osteoporosis	54.12 35.21 10.67	57.80 35.78 6.420	25.12 30.70 44.18	<0.0001

PIR, ratio of family income to poverty; ALT, alanine aminotransferase; AST, alanine aminotransferase; UACR, urinary albumin creatinine ratio; Cr/BUN, Creatinine BUN ratio; TG, triglycerides; HDL, high-density lipoprotein; TC, total cholesterol; Lumbar-Spin BMD, lumbar spine bone mineral density; Pelvis-BMD, pelvis bone mineral density; Total BMD, total bone mineral density.

As shown in **Table 2**, a positive correlation was observed between OP and the prevalence of sarcopenia. The OR were 1.90 (95% CI 1.13–3.18), 3.54 (95% CI 1.91–6.54), and 4.57 (95% CI 1.47–14.22) in Model 1, Model 2, and Model 3, respectively. In Model 1, which did not adjust for any variables, only total BMD was identified as a protective factor against sarcopenia occurrence. However, after adjusting for gender, age, BMI, and biochemical variables in Model 2, LS-BMD, pelvis-BMD, and total BMD emerged as protective factors against the development of sarcopenia.

**Table 2:** Associations between osteoporosis and the prevalence of sarcopenia.

Characteristics	Model1 OR (95%CI, P)	Model2 OR (95%CI, P)	Model3 OR (95%CI, P)
Osteopenia	0.76 (0.52,1.12) 0.160	1.08 (0.69, 1.69) 0.74	2.09 (0.93,4.73) 0.07
Osteoporosis Lumbar-Spine-BMD Pelvis-BMD Total BMD	1.90 (1.13,3.18) 0.02 0.06 (0.01,0.22) 0.06 1.00 (0.39,2.56) 0.990 0.02 (0.00,0.11) <0.001	3.54 (1.91, 6.54) <0.001 0.19 (0.04,0.84) 0.03 0.12 (0.04,0.35) <0.001 0.09 (0.01, 0.79) 0.03	4.57 (1.47,14.22) 0.01 11.82 (0.88,159.52) 0.06 4.96 (0.67,36.71) 0.11 0.06 (0.00,3.05) 0.15

Lumbar-Spin BMD, lumbar spine bone mineral density; Pelvis-BMD, pelvis bone mineral density; Total BMD, total bone mineral density.

To further elucidate the relationship between BMD and sarcopenia, we stratified BMD using quartiles to examine the potential association between BMD levels and sarcopenia across different genders and racial groups. We observed that the protection against sarcopenia increased with higher bone mineral density levels, regardless of gender and racial factors (**Table 3**).

**Table 3:** Associations between bone mineral density level and sarcopenia.

Characteristics	Q1	Q2	P	Q3	P	Q4	P
Sex							
Female	Reference	0.23 (0.17,0.30)	<0.001	0.21 (0.15,0.29)	<0.001	0.09 (0.05,0.16)	<0.001
Male	Reference	0.11 (0.09,0.14)	<0.001	0.06 (0.05,0.08)	<0.001	0.03 (0.02,0.04)	<0.001
Race							
Non_HispanicBlack	Reference	0.05 (0.03,0.10)	<0.001	0.03 (0.01,0.07)	<0.001	0.01 (0.01,0.04)	<0.001
Non_HispanicWhite	Reference	0.16 (0.11,0.22)	<0.001	0.1 (0.07,0.15)	<0.001	0.08 (0.05,0.12)	<0.001
Mexican American	Reference	0.22 (0.17,0.30)	<0.001	0.19 (0.14,0.26)	<0.001	0.08 (0.05,0.13)	<0.001
Other Hispanic	Reference	0.25 (0.12,0.51)	<0.001	0.38 (0.19,0.75)	0.01	0.28 (0.12,0.66)	0.004
Other Race	Reference	0.25 (0.15,0.44)	<0.001	0.2 (0.10,0.38)	<0.001	0.17 (0.08,0.39)	<0.001

### 3.2 The results of bidirectional MR analysis

#### 3.2.1 Stage 1: influence of osteoporosis on sarcopenia

In the initial phase, F statistics were computed for FA BMD, FN BMD, and LS BMD, resulting in values of 10.71, 33.38, and 26.41, respectively. Clearly, all values exceeded the threshold of 10, indicating that the selected instrumental variables (IVs) were robust enough to mitigate potential bias. The MR Steiger test was employed to assess the proportion of variance explained by the chosen variables for the factor analysis of BMD in the femoral neck, lumbar spine, and total hip. This information is presented in **Supplementary Table 1**.

The study investigated the impact of OP on ALM, revealing 16, 20, and 22 IVs for FA BMD, FN BMD, and LS BMD, respectively (**Supplementary Table 2**). The MR pleiotropy test identified horizontal pleiotropy in FN BMD-related IVs, while MR-PRESSO flagged several potential pleiotropic IVs for BMD (**Supplementary Table 3**). After removing outliers, the IVW results demonstrated a significant causal relationship between BMD and ALM. In summary, the majority of MR analyses supported the significant negative causal effect of OP on ALM (**Table 4**).

**Table 4:** MR estimates for the causal effect of osteoporosis on sarcopenia-related traits.

Exposure	Outcomes	No.of IVs	Heterogeneity Test Cochran’s Q (P)	MR Egger Intercept(P)	MR results		
					Method	OR (95%CI)	P
FA BMD	ALM	16	117.827 (<0.001)	5.95e-04 (0.901)	IVW	1.03 (0.999,1.062)	0.059
					Weighted median	1.026 (1.008,1.045)	0.006
					RAPS	1.026 (0.996,1.056)	0.085
					MR-PRESSO (2)	1.028 (1.008,1.049)	0.006
FN BMD	ALM	20	421.208 (<0.001)	0.028 (0.010)	IVW	1.090 (1.043,1.140)	0.001
					Weighted median	1.093 (1.056,1.131)	5.08e-07
					RAPS	1.100 (1.018,1.188)	0.016
					MR-PRESSO (8)	1.131 (1.092,1.170)	3.18e-12
LS BMD	ALM	22	245.896 (<0.001)	0.005 (0.466)	IVW	1.090 (1.043,1.140)	1.53e-04
					Weighted Median	1.069 (1.044,1.095)	2.10e-08
					RAPS	1.075 (1.049,1.101)	3.2e-09
					MR-PRESSO (8)	1.080 (1.062,1.098)	2.86e-19

FA BMD, forearm bone mineral density; FN BMD, femoral neck bone mineral density; LS BMD, lumbar spine bone mineral density; ALM, appendicular lean mass; IVW, inverse-variance-weighted; RAPS, robust adjusted profile score; MR-PRESSO, MR-pleiotropy residual sum and outlier.

#### 3.2.2 Stage 2: influence of sarcopenia on osteoporosis

In the second stage, the F statistic for ALM was calculated, yielding a value of 17.22. The proportion of variance explained by the selected IVs for ALM is provided in **Supplementary Table 1**.

The study investigated the impact of ALM on OP. A total of 560, 520, and 519 appropriate IVs were obtained for ALM, respectively (**Supplementary Table 5**). The MR pleiotropy test revealed no horizontal pleiotropy, however, MR-PRESSO detected several potential pleiotropic IVs for ALM (**Supplementary Table 3**). When combined with the above-mentioned MR results, the analyses suggested that ALM had no significant causal effect on FA BMD or FN BMD, but identified a significant negative causal effect on LS BMD, consistent with the IVW outcomes [FA BMD-related analysis: OR (95% CI) = 0.957 (0.888, 1.031), p = 0.245; FN BMD-related analysis: OR (95% CI) = 1.011 (0.965, 1.059), p = 0.650; LS BMD-related analysis: 1.088 (1.033, 1.147), p = 0.001] (**Table 5**).

**Table 5:** MR estimates for the causal effect of sarcopenia-related traits on osteoporosis.

	Outcomes	No.of IVs	Heterogeneity Test Cochran’s Q (P)	MR Egger Intercept(P)	MR results		
					Method	OR (95%CI)	P
ALM	FA BMD	562	631.596 (0.020)	0.002 (0.420)	IVW	0.957 (0.888,1.031)	0.254
					Weighted median	0.931 (0.834,1.040)	0.208
					RAPS	0.931 (0.864,1.003)	0.058
					MR-PRESSO (1)	0.951 (0.886,1.021)	0.163
ALM	FN BMD	520	877.962 (<0.001)	0.002 (0.199)	IVW	1.011 (0.965,1.059)	0.650
					Weighted median	0.981 (0.923,1.043)	0.546
					RAPS	1.003 (0.960,1.047)	0.909
					MR-PRESSO (5)	0.988 (0.947,1.170)	0.589
ALM	LS BMD	519	808.448 (<0.001)	6.41e-04 (0.646)	IVW	1.088 (1.033,1.147)	0.001
					Weighted Median	1.059 (0.990,1.133)	0.093 0.006
					RAPS	1.071 (1.020,1.124)	
					MR-PRESSO (3)	1.068 (1.018,1.121)	0.007

In Stage 2, several IVs were excluded due to significant overlap with confounding SNPs (**Supplementary Table 4**). Nevertheless, the significance of the MR analysis outcomes and the presence of horizontal pleiotropy remained unchanged (**Table 5**).

## 4 Discussion

This study employs an epidemiological cross-sectional analysis and a two-sample MR analysis to investigate the causal relationship between OP and sarcopenia risk. Our findings support a correlation between OP and sarcopenia risk, and further confirm the potential causal relationship.

Our investigation indicates a correlation between higher levels of bone mineral density (BMD) and a reduced risk of sarcopenia. However, subsequent adjustments for all variables in a multivariate logistic regression did not reveal BMD to function as an independent risk factor for sarcopenia. As a result, our conclusion is limited to recognizing BMD as a contributory factor to sarcopenia. Nevertheless, a prior study suggested a positive correlation between BMD and ALM, coupled with a negative association with fat mass. This correlation was established after meticulous control for potential confounding variables, encompassing individuals from African American, Caucasian, and Chinese demographics (31). The discrepancies in our findings could likely be attributed to variations in population demographics, dietary patterns, and levels of healthcare provision. Despite these distinctions, our overall assessment affirms that bone mineral density (BMD) serves as a significant risk factor for sarcopenia. It becomes imperative to screen individuals exhibiting abnormal BMD for potential sarcopenia, allowing for early intervention and disease management, thereby mitigating the escalation of economic burdens. Furthermore, our study highlights that osteopenia or osteoporosis (OP) independently contribute as risk factors for sarcopenia. Thus, particular attention must be directed toward preventing sarcopenia in OP patients, as this measure could significantly decrease the incidence of severe complications.

While OP and sarcopenia exhibit analogous changes, a gender disparity exists between the two conditions. Osteoporosis predominantly manifests in postmenopausal women, whereas the decline in skeletal muscle mass is more commonly observed in men (32). Our findings demonstrate a statistically significant variance in the prevalence of sarcopenia across genders. The outcomes derived from the multivariate logistic regression affirm that males exhibit a heightened risk of developing muscle atrophy compared to females (OR > 1). This observation aligns consistently with our research conclusions. Postmenopausal women often encounter a simultaneous decline in both bone and muscle mass attributed to hormonal fluctuations and the aging process (33). Skeletal muscle and bone losses were associated with the menopausal transition. A study divided 897 women into four groups: premenopause, peri-menopause, peri-menopause late, and postmenopause. The study results suggested that there was a significant linear decrease in appendicular lean mass, appendicular lean mass index, femoral neck bone density, and T score in the menopause group. Compared to postmenopausal women, premenopausal women showed higher ALM (34). An observed study revealed that women diagnosed with sarcopenia exhibited lower BMDs across all assessed sites, encompassing LS BMD, FN BMD, FM BMD, total hip, and total lean mass. In contrast, men affected by sarcopenia displayed lower BMDs solely at FN BMD and total hip sites (35). The primary rationale behind this outcome stems from the inherent hormonal disparities between men and women. Following menopause, women experience a decline in hormone levels, leading to an accelerated rate of bone loss and an increased vulnerability to bone mass reduction or osteoporosis.

Our findings underscore that OP stands as an independent risk factor for sarcopenia. Furthermore, an analysis involving 17,891 subjects from African American, Caucasian, and Chinese ethnicities revealed compelling evidence. Individuals diagnosed with sarcopenia based on ALM were found to be twice as likely to have osteopenia/osteoporosis compared to those without sarcopenia (OR = 2.04; 95% CI = 1.61, 2.60) (31). There exists an intricate and potentially causal relationship between OP and sarcopenia. An MR study unearthed compelling evidence indicating a positive causal association between BMD and fat-free mass (FFM). Specifically, BMD was found to be positively associated with FFM, including right leg FFM (β = 0.014, p-value = 0.003) and left arm FFM (β = 0.014, p-value = 0.005) (36). Another MR study identified direct causal effects wherein FN BMD, LS BMD, and FA BMD exerted a direct causal impact on ALM. Additionally, ALM exhibited a significant causal effect on LS BMD. However, no evidence supported a causal association between BMD and low grip strength. These findings align consistently with our own research, indicating a causal relationship between osteoporosis and sarcopenia. Specifically, a decrease in BMD appears to influence the loss of ALM, and a decline in ALM influences the loss of LS BMD.

Previous studies have identified osteo-sarcopenia as a pathological condition linked with aging. It is typified by porous and fragile bones, alongside sarcopenia, which entails low muscle mass and function. Termed a ‘hazardous duo,’ this condition contributes to weakened bones and an escalated fracture risk, culminating in higher mortality rates and posing a substantial global financial threat (16). Distinctive features of the aging process within the musculoskeletal system involve a gradual reduction in bone mass and the deterioration of bone microarchitecture. Simultaneously, there’s a progressive decline in muscle mass, strength, and overall functionality, culminating in the onset of osteoporosis and sarcopenia (37). There was a significant correlation observed between the reduction in muscle mass and strength and the decline in bone mass along with deteriorating bone microarchitecture (35). An increasing acknowledgment prevails regarding the interdependence of muscle and bone disorders (38), encompassing genetic regulation (39), the hormonal system (40), and intricate mechanical interactions (41). Presently, endocrine factors stand at the forefront of research endeavors aimed at unraveling the underlying pathogenesis of osteo-sarcopenia. Particular attention is directed towards myokines and osteokines, which intricately modulate communication between muscles and bones, manifesting complex effects on both tissues.

Myokines, primarily composed of peptides, represent soluble molecules secreted by muscle fibers. They regulate both biological and pathological functions of local and distant cells and organs (42). Currently, over 650 types of myokines have been identified (43). For instance, the pioneering discovery, myostatin, expressed in developing and mature muscles, negatively regulates muscle mass (44). Mutation or knockout of the myostatin (Mstn) gene leads to muscle hypertrophy, increased strength, and improved bone structure (45). Furthermore, a myokine induced by exercise, irisin, has demonstrated the capacity to enhance heat production and increase bone mass (46). Beyond these, recent discoveries have unveiled additional myokines such as fibroblast growth factor 21 (FGF21), β-aminoisobutyric acid (BAIBA), and Meteorin-like (METRNL). These myokines have been reported to influence osteogenesis, myogenesis, and the intricate bone-muscle crosstalk during the aging process.

The regulation of myostatin in myogenesis is remarkably intricate. Primarily, myostatin exerts its effects by downregulating paired box 7 (Pax7), inhibiting the activation and self-renewal of quiescent satellite cells. Additionally, in C2C12 myoblast cells, myostatin induces G1 phase arrest by promoting p21 expression while inhibiting phosphorylation of cell cycle proteins dependent on kinase 2 (CDK2) and kinase 4 (CDK4). Moreover, myostatin reduces the expression of myogenic genes by downregulating the MEK/ERK1/2 MAPK pathway and/or the AKT/mTORC1 signaling pathway (47). Furthermore, myostatin plays a multifaceted role in muscle protein translation, degradation, and synthesis (48). Overall, the mechanisms through which myostatin influences skeletal muscle communication are highly complex, warranting further in-depth exploration in this area of research.

Myostatin demonstrates a direct impact on osteocytes. *In vitro* osteocyte models have revealed that myostatin enhances the expression of Wnt pathway inhibitors, SOST and dickkopf Wnt signaling pathway inhibitor 1 (DKK1). Additionally, it stimulates the expression of the osteoclastogenesis gene, Rankl, in osteocytes (49). Notably, myostatin not only influences the expression of receptor activator of nuclear factor kappa B ligand (RANKL) in osteocytes but also directly upregulates genes associated with osteoclast differentiation, such as NFTC1 (50). Moreover, *in vitro* studies show that myostatin inhibits the expression of Alp, osteocalcin, and crucial osteoblast transcription factors, osterix, and Runx2 (50).Subsequent research involving myostatin inhibition has indicated that its suppression can restore ALP signaling and RUNX2 expression in aged primary myofibers and C2C12 cells treated with MC3T3-E1 (51). This occurs due to the myostatin inhibitor binding to myostatin, attenuating the negative impact of aged muscle on osteogenic differentiation. As aging brings about muscle and bone degeneration, the attention has shifted toward myostatin inhibitors, which exhibit a positive effect on both muscles and bones. Consequently, further investigation into myostatin inhibitors holds promise for offering novel insights into aging research.

Moreover, osteocalcin (OCN), is an osteokines, secreted protein by osteoblasts with hormone-like characteristics and physiological functions. Studies examining OCN’s effect on muscles indicate that OCN deficiency reduces activity in mice, yet injection of exogenous osteocalcin restores their activity (52). Liu S. et al. (53) demonstrated that under-carboxylated OCN enhances the proliferation and myogenic differentiation of C2C12 myoblasts through activation of the PI3K/Akt, p38, and GPRC6A-ERK1/2 signaling pathways. This suggests that OCN has the potential to improve muscle mass and strength, providing a theoretical foundation for anti-aging interventions. Thus, we strongly believe that delving deeper into the bone-muscle crosstalk pathway, particularly exploring myokines and osteokines at the molecular level, is indispensable in understanding and treating conditions such as osteoporosis (OP) and sarcopenia. This avenue of research holds significant promise for the prevention and management of these interconnected diseases.

Poor musculoskeletal health significantly escalates the risk of mortality, independent of age. In a study combining BMD and ALM groups in a model, BMD emerged as a predictor of mortality (HR 1.74, 95%CI 1.09-2.78; HR 2.82, 95%CI 1.70-4.70, respectively), while low ALM showed borderline significance (HR 1.52, 95%CI 1.00-2.31) (54). This underscores the critical significance of preventing and treating osteo-sarcopenia, particularly among the elderly. A systematic review indicates that chronic resistance training proves safe and efficacious in enhancing muscle mass, strength, and quality in older adults with sarcopenia. Moreover, it aids in increasing or maintaining bone density (55). Additionally, combining creatine with resistance training yields better muscle gain, strength, and function (56). Currently, lifestyle improvements remain the primary approach in osteo-sarcopenia treatment, with limited studies focusing on drug interventions. In future research, the development of drugs in this domain stands as an imperative challenge yet to be fully addressed.

This study has several notable strengths. Firstly, it is a large cross-sectional study utilizing NHANES data. Additionally, it employs a two-sample bidirectional Mendelian randomization analysis, with the results from both approaches strongly supporting each other, increasing the level of confidence in the findings. Cross-sectional studies can explore the relationship between OP and sarcopenia. MR overcomes the influence of numerous confounding factors and reverse causality bias in traditional studies. In epidemiological cross-sectional research, investigating causal relationships is an involved aspect. The second advantage is that in studying the relationship between OP and sarcopenia, we included BMD as an indicator. Simultaneously, we analyzed the relationship between BMD and sarcopenia. Furthermore, we conducted a stratified study on BMD and its relationship with sarcopenia. Although we ultimately found that reduced BMD is a risk factor for sarcopenia, we did not find that reduced BMD is an independent risk factor for sarcopenia. However, we observed that higher BMD levels are associated with a lower risk of developing sarcopenia. However, this study has some potential limitations. Firstly, in cross-sectional studies, there may be self-reporting and recall biases in the diagnosis of OP. The diagnosis of sarcopenia is made by adjusting BMI with ALM. Secondly, the results may be subject to bias, and they cannot be generalized beyond the scope of this data, which is limited to the United States, and may not apply to other populations or the European demographic. Because of the limitations inherent in cross-sectional studies and MR, further corroborative evidence from higher-level studies, such as randomized controlled trials and longitudinal studies, is needed.

## 5 Conclusion

Our study identified that it is a positive correlation exists between OP and the prevalence of sarcopenia. OP and SP may have a strong causal relationship. OP is more prone to losing ALM, and severe loss of ALM may lead to a decrease in LS BMD. These findings require confirmation through additional longitudinal cohort studies utilizing larger sample sizes.

## Data Availability

The datasets presented in this study can be found in online repositories. The names of the repository/repositories and accession number(s) can be found in the article/**Supplementary Material**.
