# Supplementary material for: Association between sarcopenia and osteoporosis: the cross-sectional study from NHANES 1999–2020 and a bi-directions Mendelian randomization study
Source: Front Endocrinol (Lausanne). 2024 Oct 8;15:1399936. doi: 10.3389/fendo.2024.1399936 (PMC11493612; doi:10.3389/fendo.2024.1399936)
Supplement: Supplementary file 1 [file Table1.docx]

Supplementary Table 1. Variance explained by IVs in MR-Steiger analysis.

| Exposure | outcome | snp_r2. exposure | snp_r2. outcome | MR-Steiger |
| --- | --- | --- | --- | --- |
| FA BMD | ALM | 0.057 | 3.24E-04 | TRUE |
| FN BMD | ALM | 0.031 | 1.00E-03 | TRUE |
| LS BMD | ALM | 0.038 | 1.00E-03 | TRUE |
| ALM | FA BMD | 0.126 | 8.20E-02 | TRUE |
| ALM | FN BMD | 0.115 | 2.80E-02 | TRUE |
| ALM | LS BMD | 0.115 | 3.10E-02 | TRUE |

BMD: bone mineral density; ALM: appendicular lean mass; FA: forearm; FN: femoral neck; LS: lumbar spine; IVs: instrumental variables.
