# Supplementary material for: Association between sarcopenia and osteoporosis: the cross-sectional study from NHANES 1999–2020 and a bi-directions Mendelian randomization study
Source: Front Endocrinol (Lausanne). 2024 Oct 8;15:1399936. doi: 10.3389/fendo.2024.1399936 (PMC11493612; doi:10.3389/fendo.2024.1399936)
Supplement: Supplementary file 4 [file Table4.docx]

Supplementary Table 4. The removed IVs due to confounders

| Exposures | Outcomes | The removed IVs |  |
| --- | --- | --- | --- |
| FA BMD | ALM | NA | |
| FN BMD | ALM | NA | |
| LS BMD | ALM | NA | |
| ALM | FA BMD | rs1051952, rs10807137, rs10810474, rs10849576, rs12325539, rs12714414, rs12761076, rs1291114,  rs1355603, rs1487441, rs1662842, rs17036160, rs2052478, rs2287821, rs2524139, rs2531991, rs2721940,  rs2764264, rs28678024, rs34517439, rs3764002, rs3769885, rs3814333, rs3853252, rs4076427, rs41271299,  rs42039, rs4909912, rs4985445, rs501250, rs55745410, rs57904377, rs59985551, rs7107356, rs76895963,  rs798528, rs9391254, rs963317, rs9853018 | |
| ALM | FN BMD | rs1051952, rs10807137, rs10810474, rs12325539, rs12714414, rs12761076, rs1291114, rs1355603, rs1487441,  rs1662842, rs17036160, rs2287821, rs2524139, rs2531991, rs2764264, rs28678024, rs34517439, rs3769885,  rs3814333, rs3853252, rs4076427, rs41271299, rs4909912, rs4985445, rs501250, rs55745410, rs57904377,  rs59985551, rs7107356, rs76895963, rs798528, rs9391254, rs963317, rs9853018 | |
| ALM | LS BMD | rs1051952, rs10807137, rs10810474, rs12325539, rs12714414, rs12761076, rs1291114, rs1355603,  rs1487441, rs1662842, rs17036160, rs2287821, rs2524139. rs2531991, rs2764264, rs28678024,  rs34517439, rs3769885, rs3814333, rs3853252, rs4076427, rs41271299, rs4909912, rs4985445,  rs501250, rs55745410, rs57904377, rs59985551, rs7107356, rs76895963, rs798528, rs9391254,  rs963317, rs9853018 | |

BMD: bone mineral density; ALM: appendicular lean mass; FA: forearm; FN: femoral neck; LS: lumbar spine; IVs: instrumental variables.
